# The clinicopathological significance of UBE2C in breast cancer: a study based on immunohistochemistry, microarray and RNA-sequencing data

**DOI:** 10.1186/s12935-017-0455-1

**Published:** 2017-09-25

**Authors:** Chao-hua Mo, Li Gao, Xiao-fei Zhu, Kang-lai Wei, Jing-jing Zeng, Gang Chen, Zhen-bo Feng

**Affiliations:** 1 Department of Pathology, The First Affiliated Hospital of Guangxi Medical University, 6 Shuangyong Road, Nanning, 530021 Guangxi Zhuang Autonomous Region China; 2 Department of Pathology, The Fourth Affiliated Hospital of Guangxi Medical University, Liuzhou Worker’s Hospital, 1 Liushi Road, Liuzhou, 545005 Guangxi Zhuang Autonomous Region China

**Keywords:** Breast cancer, Ubiquitin-conjugating enzyme E2C, Immunohistochemistry, Public database, Clinico-pathological significance, Gene alteration

## Abstract

**Background:**

Ubiquitin-conjugating enzyme E2C (UBE2C) has been previously reported to correlate with the malignant progression of various human cancers, however, the exact molecular function of UBE2C in breast carcinoma (BRCA) remained elusive. We aimed to investigate UBE2C expression in BRCA and its clinical significance.

**Methods:**

The expression of UBE2C in 209 BRCA tissue samples and 53 adjacent normal tissue samples was detected using immunohistochemistry. The clinical role of UBE2C was analyzed. Public databases including the human protein atlas and Oncomine were used to assess UBE2C expression in BRCA. Moreover, the cancer genome atlas (TCGA) database was employed to investigate the prognostic value of UBE2C in BRCA.

**Results:**

The positive expression rate of UBE2C in BRCA was 70.8% (148/209), and UBE2C expression in the adjacent breast tissue was negative. The expression of UBE2C was positively correlated with tumor size (r = 0.32, P < 0.001), histological grade (r = 0.237, P = 0.001), clinical stage (r = 0.198, P = 0.004), lymph node metastasis (r = 0.155, P = 0.026), HER2 expression level (r = 0.356, P < 0.001), Ki-67 expression level (r = 0.504, P < 0.001), and P53 expression level (r = 0.32, P = 0.001). Negative correlations were found between UBE2C expression and the ER (r = − 0.403, P < 0.001) and PR (r = − 0.468, P < 0.001) status. UBE2C gene expression data from the public databases all proved that UBE2C was overexpressed in BRCA. According to the TCGA data analysis, a higher positive expression of UBE2C was associated with worse survival of BRCA patients (P = 0.0428), and data from cBioPortal indicated that 11% of all sequenced BRCA patients possessed a gene alteration of UBE2C, predominately gene amplification and mRNA regulation.

**Conclusion:**

Ubiquitin-conjugating enzyme E2C might pose an oncogenic effect on the progression of BRCA.

## Background

Breast carcinoma (BRCA) is one of the most common malignant neoplasms in humans and has a high cancer-related morbidity in females, ranking 6th in mortality for females [1]. BRCA is a highly heterogeneous neoplasm [2], and the mechanism underlying its initiation and development remains unclear. Therefore, the identification of biomarkers for early diagnosis, prognosis judgment and treatment of BRCA is urgently needed. Ubiquitin-conjugating enzyme E2C (UBE2C), a crucial part of the ubiquitin-conjugating enzyme complex, is involved in the ubiquitin–proteasome system. The ubiquitin–proteasome pathway is one of the main pathways of protein degradation in eukaryotes and serves as an important component in the post-translational modification of proteins. The process of ubiquitination is associated with many biological processes [3–6]. In recent studies, the dysregulation of the ubiquitination process has been discovered to play essential roles in the occurrence and progression of cancers, and ubiquitination has, thus, become a new therapeutic target for cancer [7–9]. Previous studies have reported overexpression of UBE2C in some cancers such as colorectal carcinoma [10], pancreatic carcinoma [11], cervical cancer [12], bladder carcinoma [13], esophageal squamous cell carcinoma [9] and lung cancer, etc. [14]. Particularly, cancers with a high degree of malignancy, low differentiation and high metastatic tendency usually present with higher UBE2C expression and poor patient survival [3]. Although several studies have confirmed UBE2C overexpression in BRCA and the prognostic significance of UBE2C in BRCA [8, 15–17], the specific role and molecular mechanism of UBE2C expression in BRCA is unclear. Therefore, in this study, we employed immunohistochemistry (IHC) and bioinformatics analysis guided by public databases containing data on gene expression in cancer to detect UBE2C expression in BRCA and adjacent tissues. Moreover, we investigated the clinico-pathological significance of UBE2C expression in BRCA and endeavored to elucidate the molecular mechanism underlying it.

## Materials and methods

### IHC

#### Patient population

We collected archived wax blocks of 209 pathologically diagnosed infiltrating ductal breast carcinoma tissues and 53 corresponding adjacent tissues that were 5 cm away from the tumor edges in Liuzhou Worker’s Hospital during the period of January 2013 to March 2015. All of the patients were females ranging from 31 to 81 years old with a median age of 50 years. The clinico-pathological information of the 209 infiltrating ductal carcinoma tissue samples is listed in Table 1. Study protocol was approved by The Ethical Committee of First Affiliated Hospital of Guangxi Medical University. All the patients signed the written informed consents before participating in the study.

**Table 1:** Clinico-pathological features of the 209 cases of infiltrating ductal breast carcinoma tissues

Clinico-pathological variables	Group	Number (%)
Age (year)	≤ 50	110 (52.6)
	> 50	99 (47.4)
Histological grade	I	18 (8.6)
	II	97 (46.4)
	III	94 (45.0)
Pathological stage (_P_TNM)	I–II	145 (69.4)
	III–IV	64 (30.6)
Tumor size (cm)	≤ 2	57 (27.3)
	2–5	131 (62.7)
	> 5	21 (10.0)
Lymph node metastasis	N0	104 (49.3)
	N1	54 (25.8)
	N2	22 (10.5)
	N3	29 (13.9)
Distant metastasis	M0	204 (97.6)
	M1	5 (2.4)
Clinical stage	I	23 (11.0)
	II	122 (58.4)
	III	59 (28.2)
	IV	5 (2.4)
Molecular types	Luminal A	62 (29.7)
	Luminal B (HER2−)	33 (15.8)
	Luminal B (HER2+)	33 (15.8)
	HER2 overexpression	42 (20.1)
	Triple-negative	39 (18.7)

#### Major reagent

The rabbit anti-human monoclonal antibody against UBE2C (1:100 dilution) was purchased from the Abnova Co., ltd. Fast enzyme-labeled Goat anti-mouse/rabbit IgG polymer, citrate buffer, phosphate buffer saline (PBS) and diaminobenzidine (DAB) indicator were all purchased from Fuzhou Maixin Biotechnology Development Co., ltd. Apart from the rabbit anti-human monoclonal antibody against UBE2C, the following antibodies were used in IHC: rabbit anti-human HER2 monoclonal antibody (EP3, Fuzhou Maixin Biotechnology Development Co., ltd), rabbit anti-human P53 monoclonal antibody (SP5, Fuzhou Maixin Biotechnology Development Co., ltd), rabbit anti-human ER monoclonal antibody (SP1, Fuzhou Maixin Biotechnology Development Co., ltd), rabbit anti-human PR monoclonal antibody (SP2, Fuzhou Maixin Biotechnology Development Co., ltd) and mouse anti-human Ki67 monoclonal antibody (MIB-1, Fuzhou Maixin Biotechnology Development Co., ltd). All of the specimens were fixed with 10% neutral formaldehyde solution and embedded in paraffin. Hematoxylin–eosin staining and immunohistochemical staining were performed on 4-μm-thick sliced tissue sections. Immunohistochemistry in two-step was performed following the operating instructions of the kit to examine the expression of UBE2C, HER2, P53, ER, PR and Ki-67. The positive control for the UBE2C staining was normal human placenta tissue, and PBS instead of the first antibody was used as the blank control.

#### Evaluation of immunohistochemical staining

The positive UBE2C signal was localized to the nucleus and cytoplasm. The staining intensity was scored as follows: 0 for no staining, 1 for canary yellow, 2 for yellow and 3 for brown. The percentage of positive cells was subdivided into four groups: 0 for less than 5%, 1 for 6–25%, 2 for 26–50%, 3 for 51–75% and 4 for more than 75%. Multiplication of the two scores provided the final immunohistochemistry score. The eventual determination of the results was defined as follows: 0 for negative (−), 1–2 for weak positive (+), 3–4 for positive (++) and ≥ 5 for strong positive (+++) [18]. According to the 2013 ASCO/CAP guidelines, moderate or strong HER2 expression in 10% of the tumor cell membranes was considered as HER2 positive [19]. As for p53, strong nuclear staining in at least 10% of the tumor cells was regarded as positive [20]. A positive result for ER and PR staining was defined as positive nuclear reactivity in at least 1% tumor cells [21]. Negative and positive Ki-67 immunostaining corresponded to < 14% and ≥ 14% of the Ki-67 positive tumor cells, respectively [22]. With regard to the interpretation of all the immunostaining results, 1000 cells from ten randomly selected high-power visual fields were counted by two pathologists independently. When the difference between evaluation results from the two pathologists was more than 10%, the immunostaining results were re-evaluated.

### Bioinformational analysis of UBE2C expression from public database

To validate the IHC results, UBE2C expression in BRCA and normal tissues was obtained from the HPA database. The HPA database is a huge repository of transcriptome and proteome data generated from RNA-sequencing analysis and immunohistochemistry analysis, which reflected the important value of the HPA database in protein expression analysis [23]. In this study, we compared the UBE2C expression in BRCA samples as well as the immunohistochemical results originating from three normal patients and 20 BRCA patients.

We further explored UBE2C expression in different types of BRCA in the Oncomine database. Oncomine is a cancer microarray database that allows researchers to mine web-based data of genome-wide expression in various types of human cancers and corresponding normal tissues [24, 25]. We compared the pattern of UBE2C expression in nine major types of BRCA including ductal BRCA, lobular BRCA, mixed lobular and ductal BRCA, intra-ductal cribriform breast adenocarcinoma, male BRCA, breast phyllodes tumor, tubular BRCA and medullary BRCA. P < 0.05 and fold change > 1.5 were selected as the threshold.

Firebrowse contains large-scale omics data from multi-platforms and is an advantageous tool for analyzing cancer gene expression in human cancers [26]. In this study, the distribution of UBE2C expression in different types of cancer was analyzed from Firebrowse (http://www.firebrowse.org/viewGene.html?gene=UBE2C), and a box plot based on UBE2C expression in diversified human cancers and corresponding normal tissues was downloaded from Firebrowse.

### TCGA data analysis of UBE2C expression and its gene alteration in BRCA

#### The prognostic significance of UBE2C expression in BRCA

We also investigated the prognostic value of UBE2C expression in BRCA using survival data of 503 BRCA patients with low UBE2C expression and 503 BRCA patients with high UBE2C expression from Oncolnc (http://www.oncolnc.org/). Kaplan–Meier survival curves were created in Oncolnc to assess the impact of UBE2C expression on the prognosis of BRCA patients.

#### Gene alteration of UBE2C in BRCA tissue from cBioPortal

We used cBioPortal (http://www.cbioportal.org) to inquire into the gene alteration status of UBE2C in BRCA [27]. The OncoPrint schematic was constructed in cBioPortal (TCGA provisional) to directly reflect all types of alterations such as amplification, deep deletion, mRNA up-regulation, and mRNA down-regulation in the UBE2C gene from 1098 BRCA patients. Alteration frequency of the UBE2C gene in BRCA tissues from different sources was visualized in a histogram comparing the distribution of UBE2C gene alterations in different subtypes of BRCA. Additionally, Kaplan–Meier survival curves were drawn in cBioPortal to evaluate the influence of gene alterations of UNE2C on the overall and disease-free survival of the 1098 BRCA patients. To achieve a comprehensive understanding of the UBE2C-centered gene regulation network, the gene network of the UBE family and the neighboring genes was generated in cBioPortal for the analysis of the interaction between these genes.

### Statistical analysis for IHC

We conducted χ^2^ tests to assess the expression of UBE2C in BRCA and para-carcinoma tissues as well as the relationship between UBE2C expression and the clinico-pathological variables of BRCA. Correlation analysis was performed by using the Spearman correlation test. Kaplan–Meier survival curves were drawn to compare the survival rates between UBE2C-positive BRCA cases and UBE2C-negative BRCA cases. Multivariable Cox hazard regression analysis was performed to assess the impact of clinico-pathological variables on the prognosis of BRCA. P < 0.05 was considered statistically significant. All of the statistical analyses stated above were performed in SPSS v.21.0

## Results

### IHC

#### The difference in UBE2C expression between BRCA and adjacent tissues

**Fig. 1:**
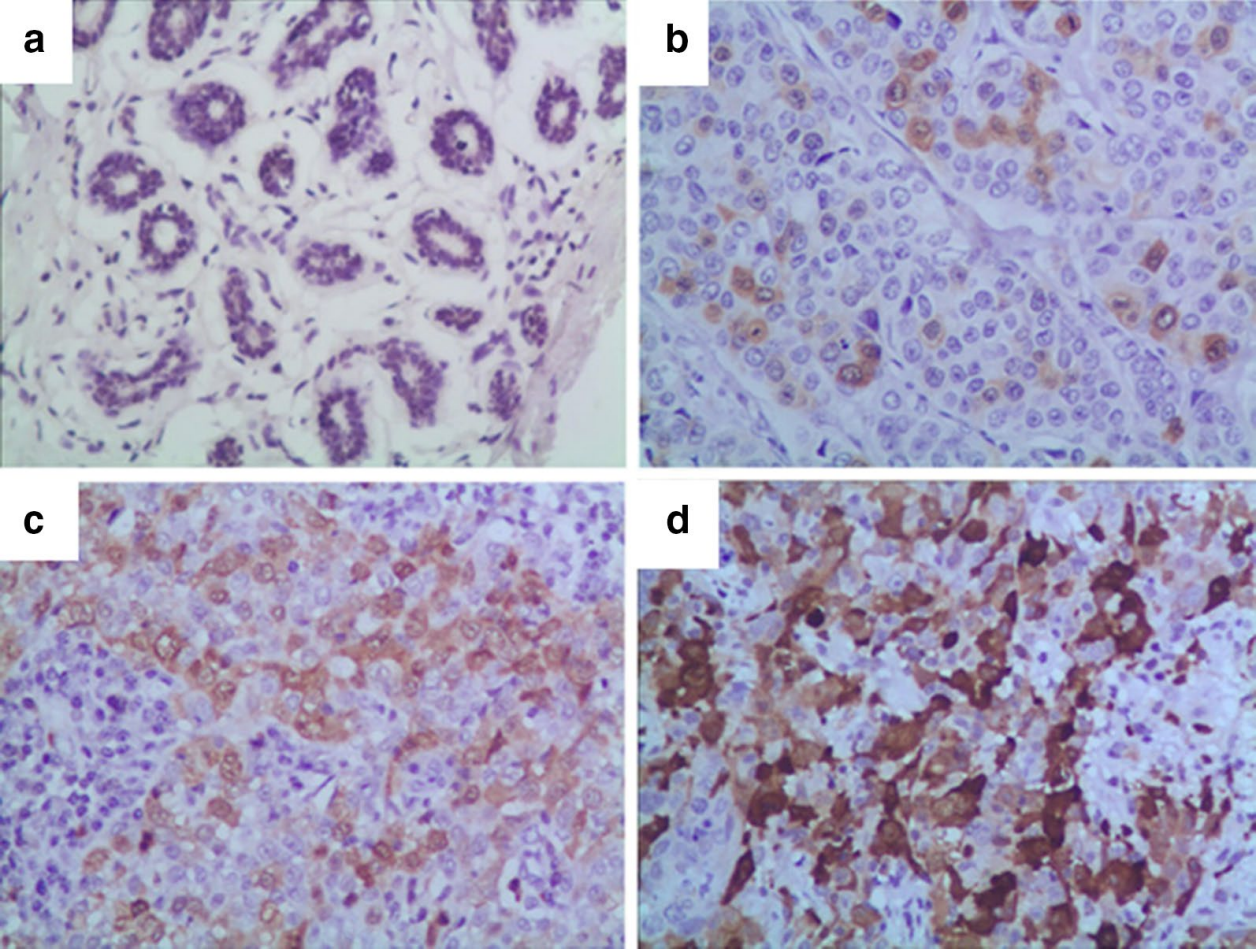
UBE2C expression in BRCA and adjacent tissues, **a** UBE2C presented negative staining in adjacent tissues; **b** UBE2C presented weak positive staining (+) in BRCA tissues; **c** UBE2C presented positive staining (++) in BRCA tissues; **d** UBE2C presented strong positive staining (+++) in BRCA tissues

The clinical pathological features of all the 209 BRCA patients were summarized in Table 1. UBE2C achieved a positive staining rate of 70.8% (148) of the 209 BRCA samples. Among the positively stained cases, there were 57, 67 and 24 cases of weak positive staining, positive staining, and strong positive staining, respectively. UBE2C-negative staining was found in the remaining 61 BRCA tissues. Conversely, all of the adjacent tissues presented negative UBE2C staining. Thus, UBE2C expression was remarkably higher in BRCA tissues than in adjacent tissues. The difference in UBE2C expression between BRCA and adjacent tissues was statistically significant (P < 0.05) (Table 2, Fig. 1).

**Table 2:** UBE2C expression between BRCA and adjacent tissues

Group	Cases	UBE2C staining		χ^2^	P value
		Negative (%)	Positive (+ to +++) (%)		
BRCA tissues	n = 209	61 (29.2)	148 (70.8)	86.256	0.00
Adjacent tissues	n = 53	53 (100.0)	0 (0.0)		

χ^2^ test was conducted to assess the expression of UBE2C in BRCA and adjacent tissues

*BRCA* breast carcinoma

#### The association between UBE2C expression in BRCA and the clinico-pathological features of BRCA

UBE2C expression in BRCA was significantly related to histological grade, tumor size, lymph node metastasis and clinical stage (P < 0.05). However, there was no significant relationship between UBE2C expression in BRCA and the age or distant metastasis of cancer (P > 0.05) (Table 3).

**Table 3:** Relationship between UBE2C expression and the clinico-pathological features of BRCA

Clinico-pathological parameters	UBE2C		χ^2^	P value
	Negative (−) %	Positive (+ to +++) %		
Age (year)			0.412	0.521
≤ 50	30 (27.3)	80 (72.7)		
> 50	31 (31.3)	68 (68.7)		
Histological grade			12.333	<0.001
I + II	45 (39.1)	70 (60.9)		
III	16 (17.0)	78 (83.0)		
Pathological stage (PTNM)			8.209	0.005
I + II	51 (35.2)	94 (64.8)		
III + IV	10 (15.6)	54 (84.4)		
Tumor size (cm)			4.367	0.042
≤ 5	59 (31.4)	129 (68.6)		
> 5	2 (9.5)	19 (90.5)		
Lymph node metastasis (N)			4.978	0.026
No (N0)	38 (35.9)	66 (64.1)		
Yes (N1–N3)	23 (21.9)	82 (78.1)		
Distant metastasis (M)			0.209	1.000
M0	60 (29.4)	144 (70.6)		
M1	1 (20.0)	4 (80.0)		
Clinical stage			8.209	0.004
I + II	51 (35.2)	94 (69.4)		
III + IV	10 (15.6)	54 (84.4)		

χ^2^ test was conducted to evaluate the correlation between UBE2C expression and the clinico-pathological parameters of BRCA

#### UBE2C expression in different molecular types of BRCA

As shown in Table 4, the UBE2C-positive expression rates were 29.0% (18/62), 72.7% (24/33), 90.9% (30/33), 95.2% (40/42) and 92.3% (36/39) in Luminal A BRCA, Luminal B (HER2−) BRCA, Luminal B (HER2+) BRCA, HER2-overexpression BRCA and triple-negative BRCA, respectively. The statistical results suggested that the positive expression rate of UBE2C was the highest in HER2-overexpression BRCA tissue, followed by triple-negative BRCA tissue, while the expression rate of UBE2C was the lowest in Luminal A BRCA tissue. There was a significant difference between UBE2C expression in various molecular types of BRCA (P < 0.05) (Table 4).

**Table 4:** Relationship between the UBE2C expression and molecular typing of breast cancer

Molecular types	UBE2C		χ^2^	P value
	Negative	Positive		
Luminal A	44 (71.0)	18 (29.0)	79.714	0.000
Luminal B (HER2−)	9 (27.3)	24 (72.7)		
Luminal B (HER2+)	3 (9.1)	30 (90.9)		
HER2 overexpression	2 (4.8)	40 (95.2)		
Triple-negative	3 (7.7)	36 (92.3)		

χ^2^ test was conducted to evaluate the correlation between UBE2C expression and molecular typing of breast cancer

#### Spearman correlation test

Correlation analyses revealed that the expression level of UBE2C in BRCA was positively associated with the following factors: tumor size (r = 0.32, P = 0.000), histological grade (r = 0.237, P = 0.001), clinical stage (r = 0.198, P = 0.004), lymph node metastasis (r = 0.155, P = 0.026), HER2 expression (r = 0.356, P = 0.000), Ki-67 expression (r = 0.504, P = 0.000) and P53 expression (r = 0.32, P = 0.001). Additionally, a negative correlation was found between UBE2C expression in BRCA and estrogen (ER) (r = − 0.403, P = 0.000) and progesterone (PR) (r = − 0.468, P = 0.000) expression.

#### The influence of UBE2C expression on BRCA patient prognosis

The follow-up period began at the operation time and ended on April 16, 2016. The end time for the dead patients was the time of death. The longest follow-up time was 1183 days, and the shortest follow-up time was 37 days. There were completed data from 8 patients that died of BRCA and censored data from 201 cases, including 184 patients still alive at the end of the follow-up period and 17 cases lost to follow-up. Among the 209 BRCS cases, there were 61 UBE2C-negative BRCA samples and two deaths. The mean survival time was 1155.316 days with a 95% confidence interval of 1117.394 to 1193.235 days. The survival curves showed that there was no significant difference between the survival rates of the two groups of patients with different UBE2C expression (P = 0.738, data not shown). Further multivariable Cox hazard regression analysis indicated no obvious effect of UBE2C on the prognosis of BRCA patients (HR = 0.535, 0.055–5.169, P = 0.589) (Table 5).

**Table 5:** Multivariable Cox hazard regression analysis for prognostic factors

	Subset	Hazard ratio (95% confidence interval)	*P* value
Age	≤ 50/> 50	1.367 (0.304–6.146)	0.683
Histological grade	I + II/III	0.786 (0.173–3.575)	0.756
Pathological stage	I + II/III + IV	0.294 (0.035–2.431)	0.256
Tumor size	≤ 5/> 5	0.789 (0.139–4.494)	0.790
Lymph node metastasis	No/yes	0.359 (0.025–5.120)	0.450
Distant metastasis	M0/M1	624,250.217 (0.000–)	0.991
UBE2C	Negative/positive	0.535 (0.055–5.169)	0.589

The upper limit of the hazard ratio for the group of distant metastasis cannot be calculated by multivariable Cox hazard regression analysis

### Bioinformational analysis of UBE2C expression from public database

**Fig. 2:**
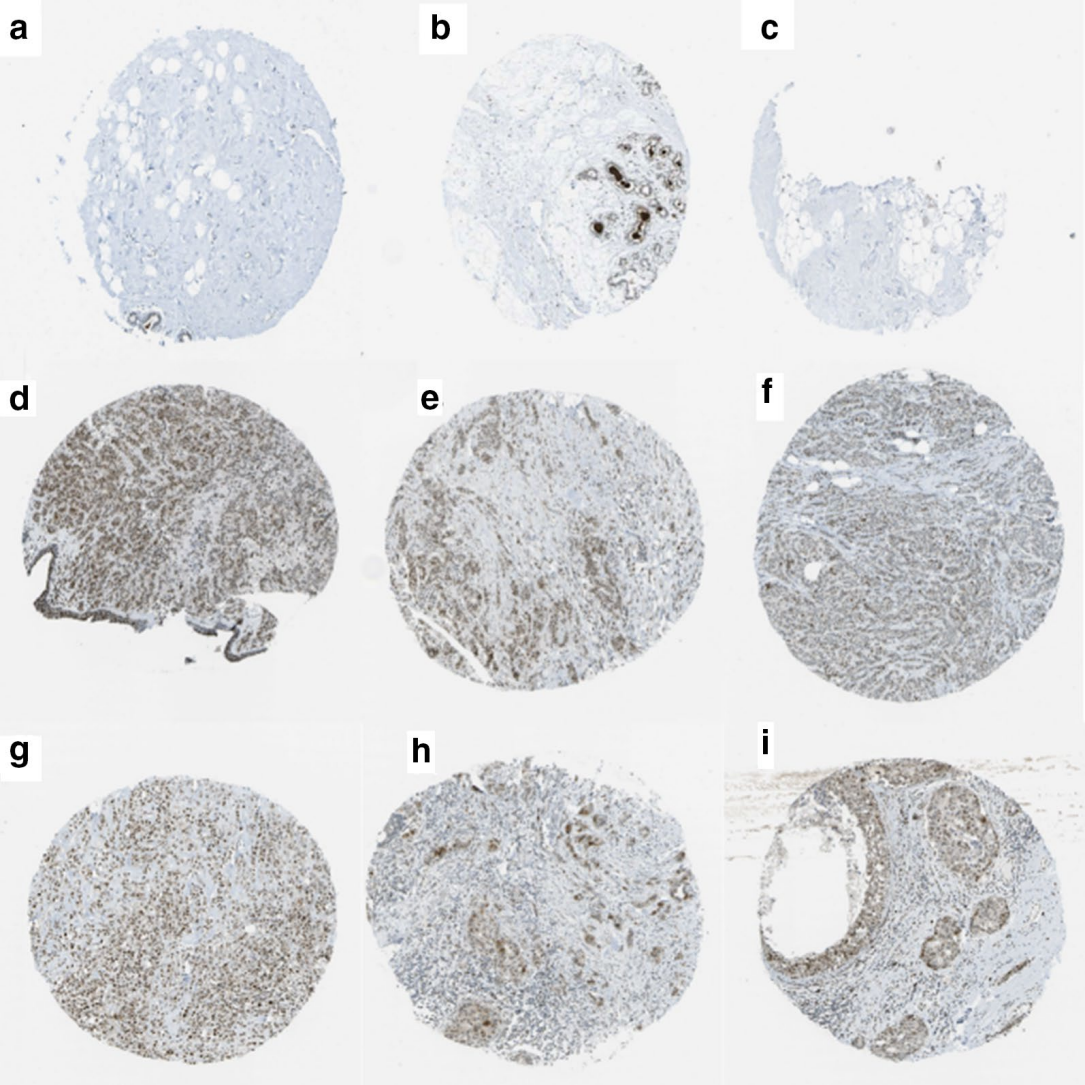
UBE2C expression in BRCA tissues and normal tissues from the HPA database. Immunohistochemical staining revealed that UBE2C exhibited low expression in three normal breast tissue samples (**a**–**c**) and high expression in 20 BRCA tissues (**d**–**i**)

According to data from the HPA database, UBE2C exhibited low expression in three cases of normal breast tissue and high expression in 20 samples of BRCA tissue. The immunohistochemical staining of UBE2C expression in normal breast tissues and BRCA tissues are displayed in Fig. 2.

**Fig. 3:**
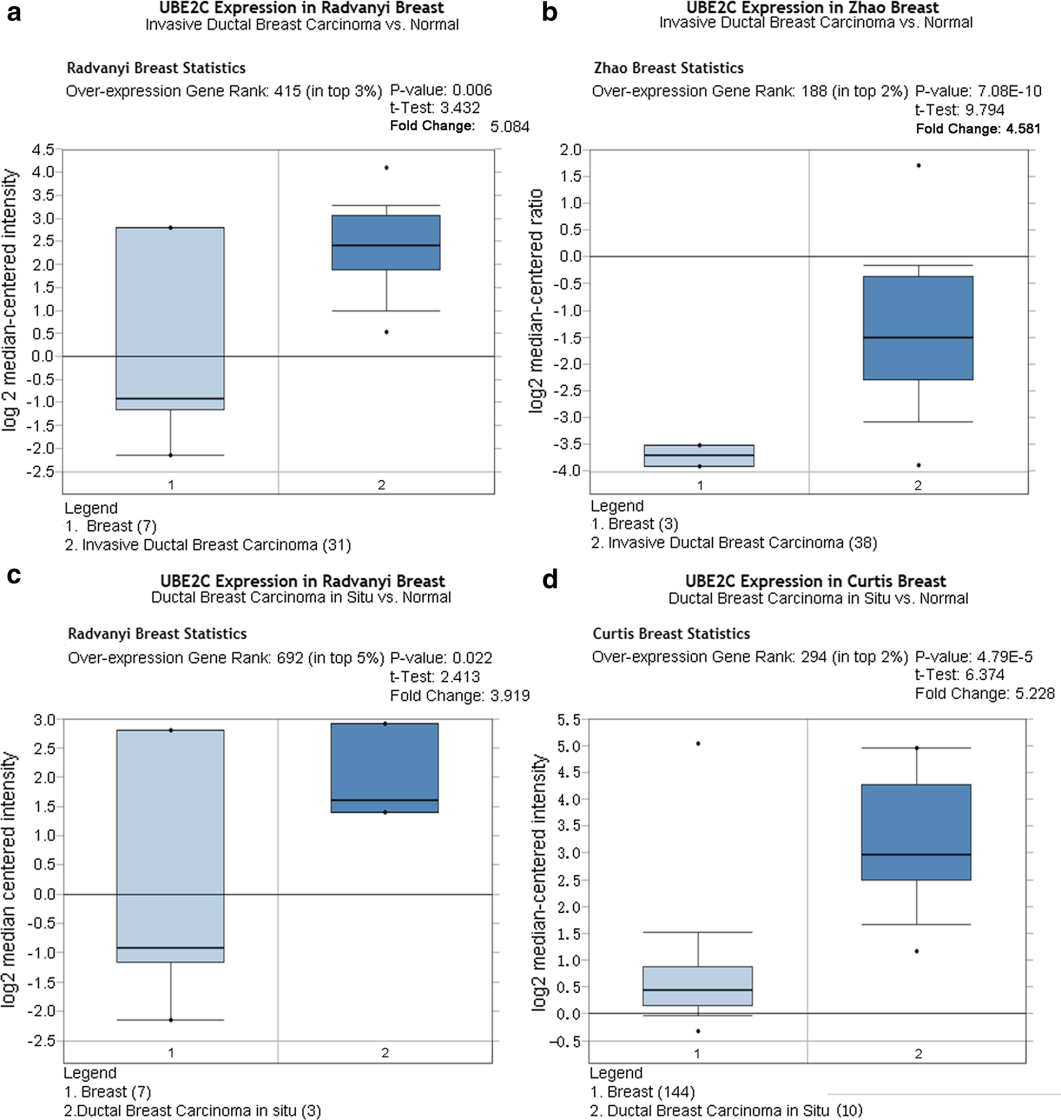
UBE2C expression in subtypes of ductal BRCA and normal tissues from Oncomine. The contrast between UBE2C expression in invasive ductal BRCA and normal breast tissues was more obvious (P = 0.006, P = 7.08E−10, **a**, **b**) than that in ductal BRCA in situ and the matched normal tissues (P = 0.022, P = 4.79E−5, **c**, **d**)

**Fig. 4:**
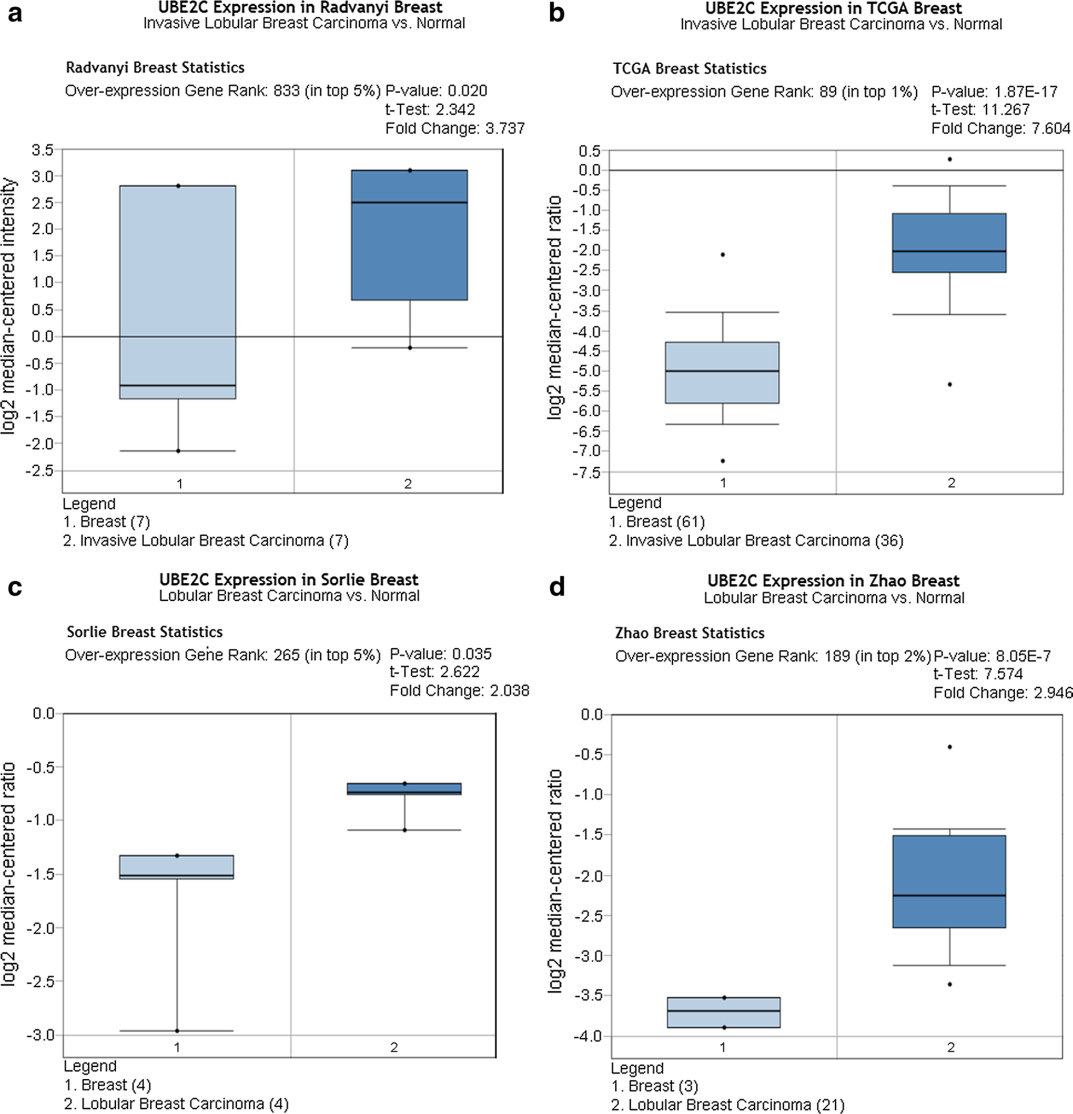
UBE2C expression in lobular BRCA and normal tissues from Oncomine. Compared with the difference in UBE2C expression between lobular BRCA tissues and normal tissues (P = 0.035, **c**; P = 8.05E−7, **d**), the difference between UBE2C expression in invasive lobular BRCA and normal tissues was more significant (P = 0.020, **a**; P = 1.87E−17, **b**)

**Fig. 5:**
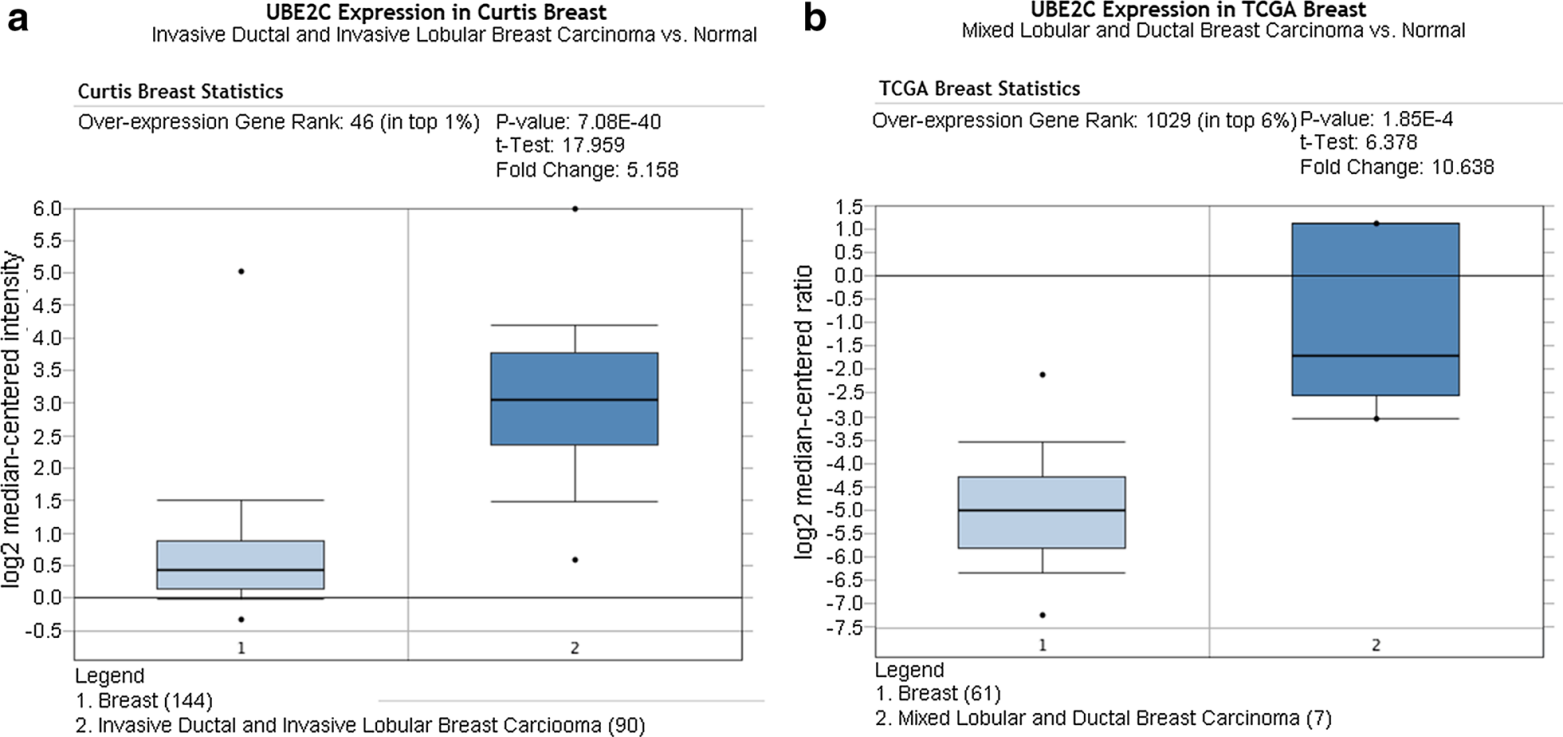
UBE2C expression in mixed ductal and lobular BRCA and normal tissues from Oncomine. The difference between UBE2C expression in invasive mixed ductal and lobular BRCA tissues and normal tissues was more significant (P = 7.08E−40, **a**) than that between mixed ductal and lobular BRCA and normal tissues (P = 1.85E−4, **b**)

**Fig. 6:**
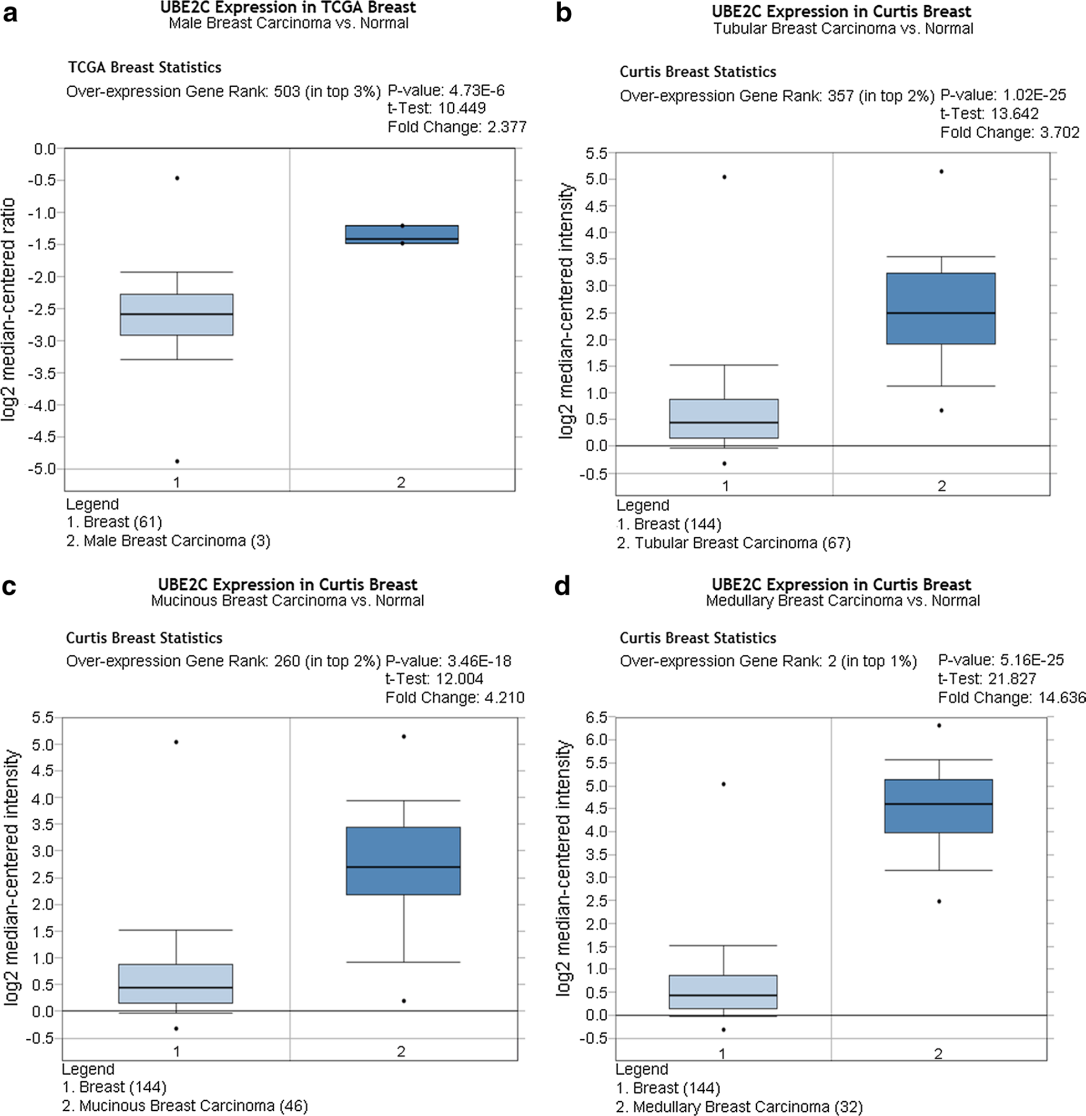
UBE2C expression in other types of BRCA and normal tissues from Oncomine. UBE2C expression was remarkably higher in male BRCA (P = 4.73E−6, **a**), tubular BRCA (P = 1.02E−25, **b**), mucinous BRCA (P = 3.46E−18, **c**) and medullary BRCA (P = 5.16E−25, **d**)

**Fig. 7:**
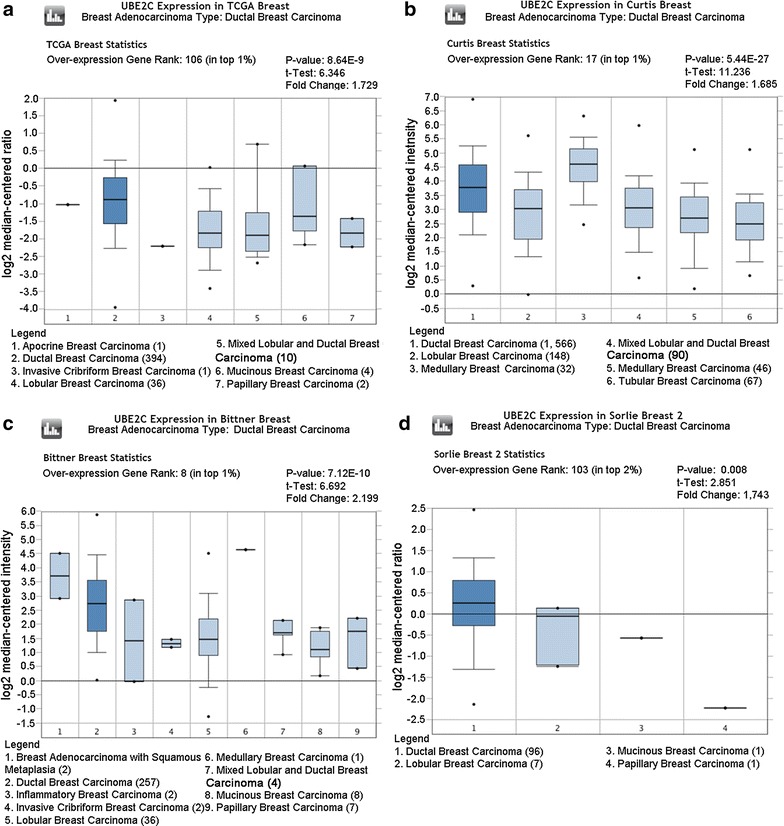
UBE2C expression in ductal BRCA and other types of breast cancer from oncomine. UBE2C expression was remarkably higher in ductal BRCA than other types of breast cancer from oncomine (P = 8.64E−9, **a**; P = 5.44E−27, **b**; P = 7.12E−10, **c**; P = 0.008, **d**)

As shown in Figs. 3, 4, 5, and 6, there was a general trend of higher UBE2C expression in all types of BRCA tissues than in the paired normal tissues (all P < 0.05). Specifically, the difference in UBE2C expression between cancer tissues and the paired normal tissues was more significant with the increased malignancy of the same type of BRCA. For example, the contrast between UBE2C expression in invasive ductal BRCA and normal breast tissues was more obvious (P = 0.006, P = 7.08E−10, Fig. 3a, b) than that between ductal BRCA in situ and the matched normal tissues (P = 0.022, P = 4.79E−5, Fig. 3c, d). A similar pattern of UBE2C expression in BRCA and normal tissues was also observed in lobular BRCA and mixed ductal and lobular BRCA (Figs. 4, 5). Moreover, UBE2C expression was remarkably higher in other types of BRCA such as male BRCA (P = 4.73E−6), tubular BRCA (P = 1.02E−25), mucinous BRCA (P = 3.46E−18) and medullary BRCA (P = 5.16E−25) (Fig. 6) compared with that in normal breast tissues. In addition, we compared UBE2C expression in ductal carcinoma and other types of breast cancer. UBE2C expression in ductal carcinoma was higher than most other types of breast cancer (Fig. 7a–d, all P < 0.05) except breast adenocarcinoma with squamous metoplasla and medullary breast carcinoma.

**Fig. 8:**
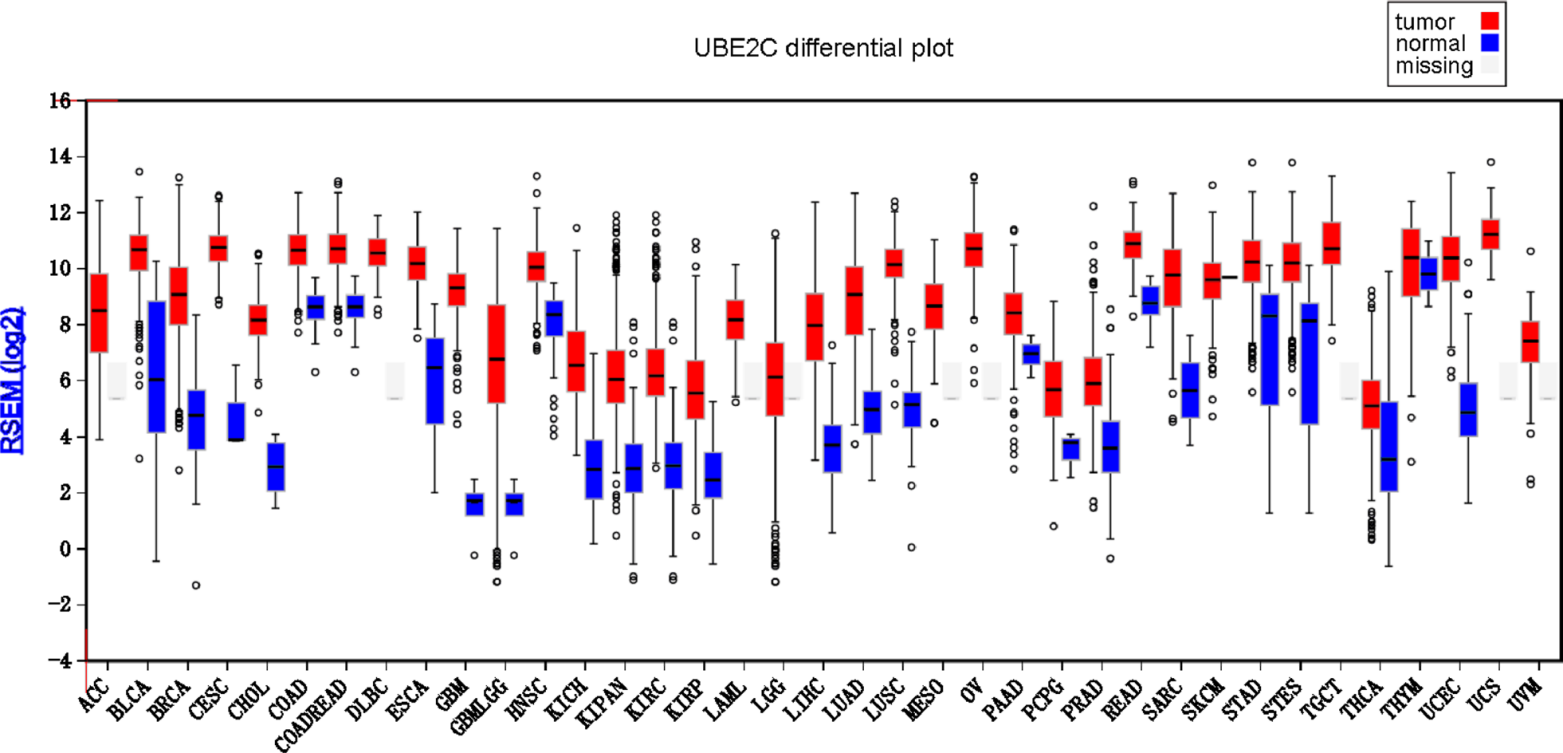
UBE2C expression in different human cancers from Firebrowse. The box plot downloaded from Firebrowse illustrates that UBE2C expression was universally higher in most human cancers, including BRCA, than in normal tissues

The box plot in Fig. 8 from Firebrowser illustrates that UBE2C expression was universally higher in most human cancers, including BRCA, than in normal tissues.

### TCGA data analysis of UBE2C expression and its gene alteration in BRCA

#### The prognostic significance of UBE2C expression in BRCA

**Fig. 9:**
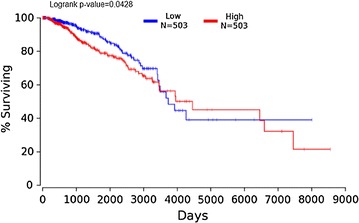
Kaplan–Meier survival analysis for UBE2C expression in BRCA patients from Oncolnc. Kaplan–Meier survival analysis of data from Oncolnc indicated that BRCA patients with lower UBE expression had a better prognosis than BRCA patients with higher UBE2C expression (P = 0.0428)

Kaplan–Meier survival analysis of data from Oncolnc revealed that BRCA patients with lower UBE2C expression had a better prognosis than BRCA patients with higher UBE2C expression (P = 0.0428) (Fig. 9).

#### Gene alteration of UBE2C in BRCA tissue from cBioPortal

**Fig. 10:**
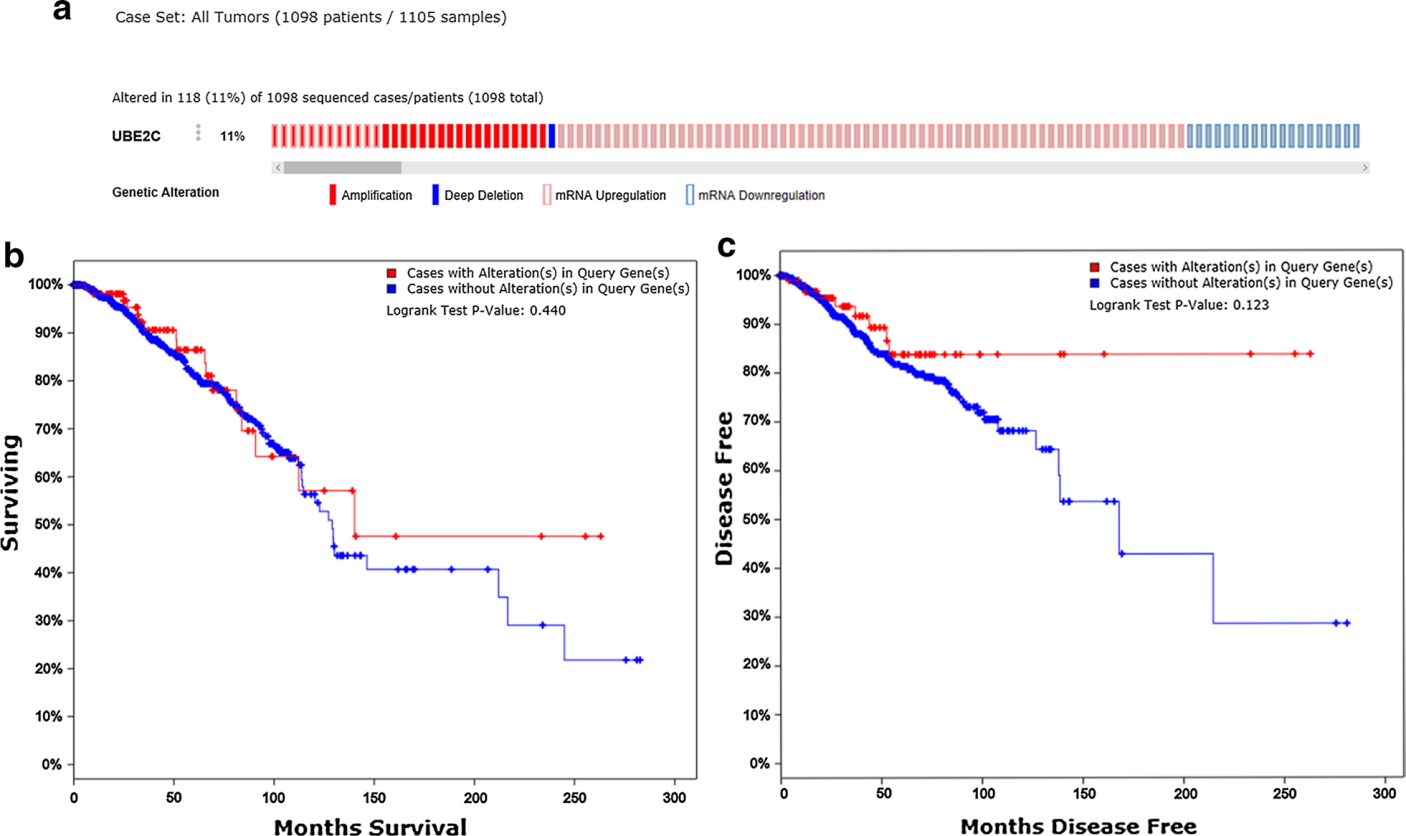
Gene alteration of UBE2C in BRCA. **a** The OncoPrint schematic showed that gene alteration of UBE2C occurred in 118 (11%) of all 1098 sequenced cases, including 30 cases of amplification, one case of deep deletion, 19 cases of mRNA down-regulation, 80 cases of mRNA up-regulation and 12 cases of a mixed type of amplification and mRNA up-regulation; Kaplan–Meier survival curves for overall survival and disease-free survival showed that there was no significant correlation between gene alteration of UBE2C and the overall survival (**b**) as well as the disease-free survival (**c**) of BRCA patients

**Fig. 11:**
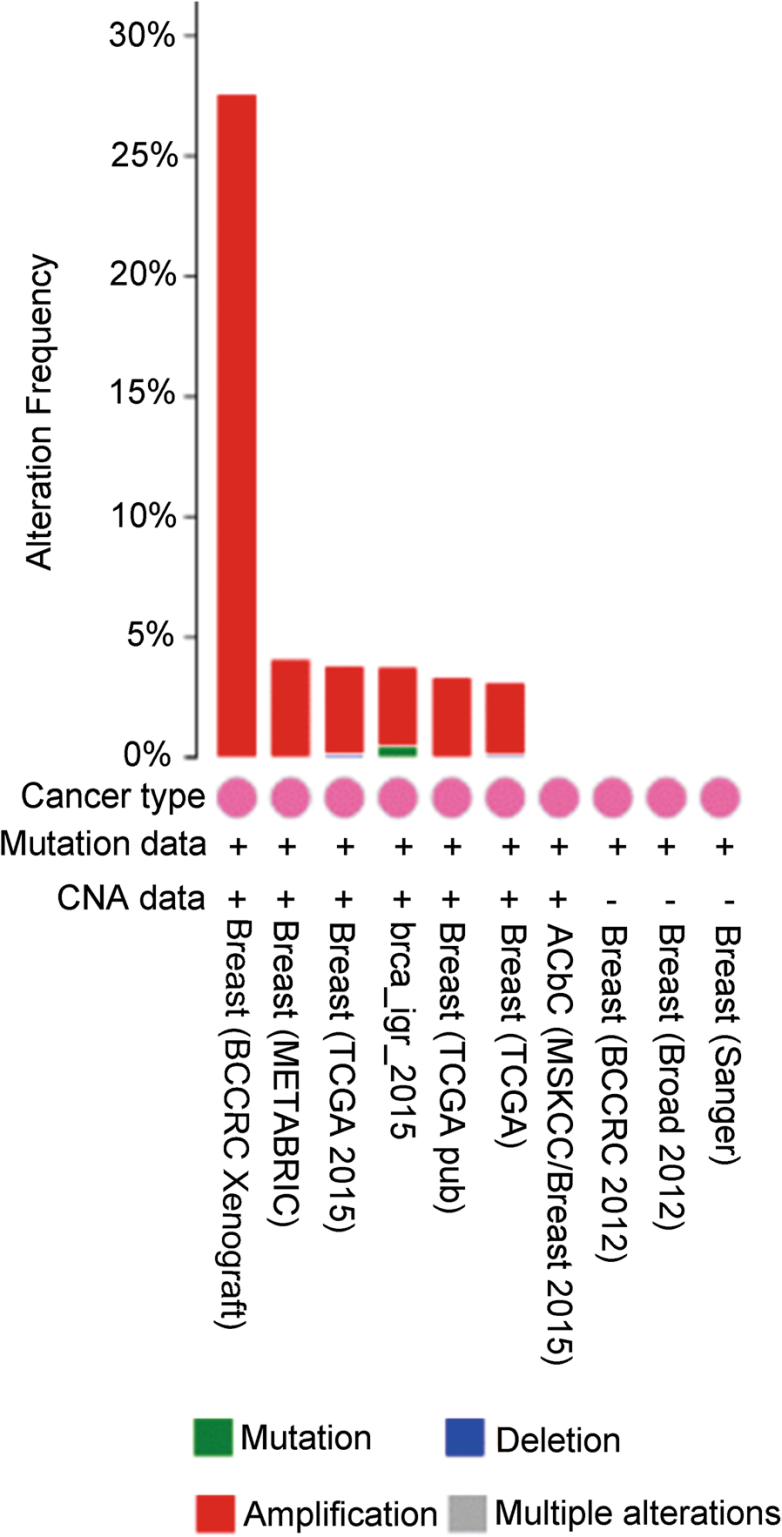
Gene alteration frequency of UBE2C in BRCA tissues from different tissues. There was less than 5% frequency of UBE2C gene alteration in most BRCA tissues except BRCA tissues from BRCACRC xenografts. Among the BRCA cases with gene alteration of UBE2C, amplification occupied the overwhelming majority of alteration types

**Fig. 12:**
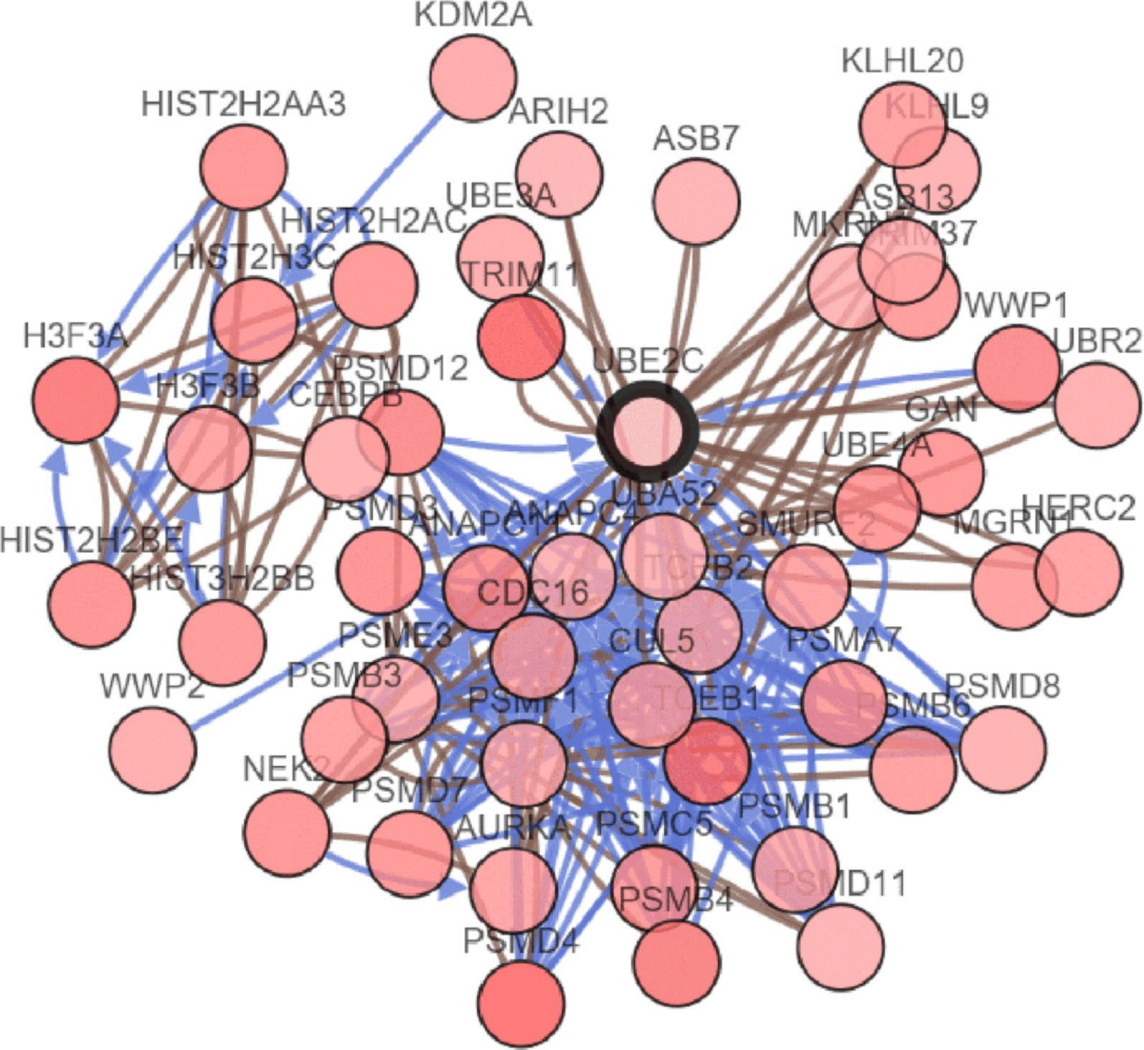
Gene regulation network from cBioPortal. The gene network generated from cBioPortal was comprised of 51 nodes representing UBE2C and the 50 most frequently altered neighboring genes. When two genes form a complex, a brown line connects the two nodes. Blue arrows between the nodes represent the interactions between them

From the OncoPrint schematic, gene alteration of UBE2C was shown to occur in 118 (11%) of all the 1098 sequenced cases, which included 30 cases of amplification, one case of deep deletion, 19 cases of mRNA down-regulation and 80 cases of mRNA up-regulation. Specifically, a mixed type of amplification and mRNA up-regulation were observed in 12 cases (Fig. 10a). Statistical results of the gene alteration frequency indicated that a UBE2C gene alteration occurred in than 5% of most BRCA tissues, except for BRCA tissues from BRCACRC xenografts (Fig. 11). Among the BRCA cases with gene alteration of UBE2C, amplification occupied the overwhelming majority of alteration types (Fig. 11). We further evaluated the relationship between gene alteration of UBE2C and the survival of BRCA patients. However, both Kaplan–Meier survival curves for overall survival and disease-free survival showed that there was no significant correlation between gene alteration of UBE2C and the overall survival or the disease-free survival of BRCA patients (Fig. 10b, c). To shed light on the underlying mechanism of UBE2C expression in BRCA, a gene regulation network containing UBE2C and the 50 most frequently altered neighboring genes was constructed. As illustrated by the network, part of the 50 most frequently altered neighboring genes such as ASB7, ARIH2, and KLHL20 form a complex with UBE2C. Some other genes including PSMD12, TRIM11, SMURF2 and PSMD8 interact directly with UBE2C (Fig. 12).

## Discussion

Breast carcinoma is one of the most common malignant neoplasms that pose a serious threat to women’s life and health. It is of great importance to delve into the pathogenesis of BRCA and identify molecular biomarkers for BRCA with high sensitivity and specificity. UBE2C, as a crucial member of the ubiquitin-conjugating enzyme family (E2), plays a pivotal role in the ubiquitin–proteasome proteolytic (UPP) pathway. Disorder of the UPP pathway initiates abnormal degradation of proteins encoded by some oncogenes and tumor suppressor genes, subsequently leading to abnormal accumulation of these proteins in the body. Therefore, the UPP system is closely related to the occurrence and progression of cancers [28, 29]. With respect to the relationship between UBE2C and BRCA, Parris et al. observed higher expression of UBE2C in invasive BRCA tissues than in normal breast tissues [15]. The prognostic value of UBE2C has also been validated to be significant in high-risk early BRCA and node-positive BRCA samples [16, 17]. In the research conducted by Rawat et al., the suppression of UBE2C could inhibit growth of BRCA cells and sensitized breast cancer cells to radiation, doxorubicin, tamoxifen and letrozole [30]. Despite the above findings, the exact function and molecular basis of UBE2C in BRCA has remained elusive. We have, for the first time, comprehensively investigated the protein expression and gene alteration of UBE2C in BRCA as well as the clinico-pathological significance of UBE2C in BRCA using a combination of immunohistochemistry (IHC) and excavation of gene expression data from public databases.

To test the hypothesis that UBE2C exerts a carcinogenic influence on BRCA, we first detected UBE2C expression in BRCA and matched normal tissues by using IHC. The results from our immunohistochemistry experiments showed that highly positive UBE2C expression in BRCA tissues contrasted sharply with the negative UBE2C expression in adjacent tissues. In addition, the expression of UBE2C was positively correlated with histological grade, tumor size, clinical stage and lymph node metastasis of BRCA. In vitro experiment in previous articles have also proved that UBE2C could enhance the proliferative, viability and invasive capacity through MTT, colony formation assay and invasion assays in lung cancer and prostate cancer [31, 32], which increased the reliability of our result that UBE2C exerted an oncogenic influence on breast cancer cells. There was also a difference between UBE2C expression in different molecular subtypes of breast cancer, among which the positive rate of UBE2C expression in HER2-overexpression BRCA was the highest, while the positive expression rate of UBE2C was the lowest in Luminal A BRCA. In the analysis of the relationship between UBE2C expression and other common biomarkers for BRCA, UBE2C expression was positively correlated with the expression of P53, HER2 and Ki-67. Conversely, UBE2C expression was negatively correlated with ER and PR expression. Research conducted by Pan et al. confirmed the overexpression of UBE2C in BRCA tissues as well as the significant correlation between UBE2C expression and the degree of cell differentiation, molecular types, and Ki-67 or HER2 expression [33]. Similarly, Berlingier et al. also reported that the expression of UBE2C in BRCA tissues was significantly higher than that in normal breast tissues, and UBE2C expression was positively correlated with Ki-67 and HER2 expression (P < 0.05) [34], which agreed with our results. With regard to the prognostic value of UBE2C expression in BRCA, a study from Psyrri et al. demonstrated that BRCA patients with high UBE2C mRNA expression experienced worse overall survival [16]. Furthermore, a Cox multivariate regression analysis from the study of Psyrri et al. suggested that UBE2C mRNA expression was an independent prognostic factor for BRCA patients [16], which was in accordance with the results from the Kaplan–Meier survival analysis for BRCA patients with different UBE2C expression in Oncolnc. Nevertheless, the Kaplan–Meier survival analysis and multivariate Cox hazard regression analysis in our study from IHC data yielded contradictory results that there was no statistically significant difference between UBE2C expression and the survival of BRCA patients. We considered that the limited patient samples and short follow-up time might contribute to the contradictory results. There were only eight patients that died during the follow-up time before April 16, 2016, which might have had a significant impact on the results. Further studies with larger patient cohorts and longer follow-up time were necessary to assess the prognostic significance of UBE2C on survival of BRCA patients.

Apart from the IHC data, UBE2C gene expression data from public databases including the HPA database, Oncomine and Firebrowse provided a consistent result showing UBE2C overexpression in BRCA tissues, which supported the IHC results. Specifically, we demonstrated an increasing significance in the difference between UBE2C expression in BRCA tissues and normal tissues with the enhanced malignancy of the same type of BRCA using data from the Oncomine database. We speculated that UBE2C expression was indicative of the malignant progression of BRCA. Moreover, the result that UBE2C expression in ductal carcinoma was obviously higher than most other types of breast cancer suggested that UBE2C could be used as a potential marker for distinguishing ductal carcinoma from other types of breast cancers.

To facilitate a comprehensive understanding of the mechanism of UBE2C expression in BRCA, we investigated gene alteration of UBE2C in BRCA with available data from cBioPortal. From the BRCA cases with UBE2C gene alteration, amplification and mRNA up-regulation comprised the major types of gene alteration, which might be triggered by the aberrant expression of upstream molecules that moderate the expression of target mRNAs. MicroRNA (miRNA), a short non-coding RNA 21–24 nucleotides in length that silences target mRNA expression through complete or incomplete binding to the 3′-untranslated region (3′-UTR) of the target mRNAs [35–43], is an typical example of a molecule that regulates downstream mRNA expression. Recently, accumulating studies have noted that miRNAs were engaged in a series of biological processes including proliferation, diversification, metastasis, and apoptosis in the development of cancer [44–52]. Several studies have also revealed that miRNAs interact with UBE2C to affect the progression of cancers. In gastric cancer, miRNA-17/20 promoted gastric cancer cell growth via targeting UBE2C [53]. In BRCA, the study of Han et al. identified UBE2C as a direct and functional target of miR-196a which can upregulate the expression of UBE2C [54]. Meanwhile, we obtained a list of miRNAs including miR-671, miR-615, miR-20a, miR-17 and miR-196a through the gene-miRNA targets function of miRWalk 2.0, which were predicted to regulate the expression of UBE2C. Thus, we hypothesized that UBE2C might serve as a substrate of specific miRNAs that stimulate the overexpression of UBE2C and exert an oncogenic influence on BRCA. Future studies were needed to investigate the role for microRNAs in regulation of UBE2C expression. To trace the origins of the UBE2C gene alteration, we explored the interactions between UBE2C and the top 50 frequently altered neighboring genes. From the gene network, some genes such as ASB7, ARIH2, KLHL20, KLHL9, ASB13, HERC2 and MGRN1 can be seen to form a complex with UBE2C; some other genes, including TRIM11, PSMD12, WWP2, SMD3 and PSMD8, directly interact with UBE2C. We hypothesized that the gene alteration of UBE2C might be explained by the abnormal activities of these genes, and these genes may also exert carcinogenic effects in BRCA through cooperating with UBE2C. Further studies are warranted to validate the interaction between UBE2C and these genes in the tumorigenesis of BRCA.

## Conclusion

In summary, overexpressed UBE2C plays a crucial role in the occurrence and development of BRCA. The clinico-pathological significance of UBE2C in BRCA suggests the possibility of using UBE2C as a worthwhile biomarker for BRCA in clinical applications. However, future experiments are necessary to unveil the molecular basis of UBE2C expression in BRCA.

## Abbreviations

UBE2C: ubiquitin-conjugating enzyme E2C
BRCA: breast carcinoma
IHC: immunohistochemistry
HPA: human protein atlas
TCGA: the cancer genome atlas
PBS: phosphate buffer saline
ER: estrogen
PR: progesterone
UPP: ubiquitin–proteasome proteolytic
MiRNA: microRNA

## References

[1] Chen W, Zhen R (2015). Incidence, mortality and survival analysis of breast cancer in China. Chin J Clin Oncol.

[2] Stricker TP, Brown CD (2017). Robust stratification of breast cancer subtypes using differential patterns of transcript isoform expression. PLoS Genet.

[3] Hao Z, Zhang H, Cowell J (2012). Ubiquitin-conjugating enzyme UBE2C: molecular biology, role in tumorigenesis, and potential as a biomarker. Tumour Biol J Int Soc Oncodev Biol Med.

[4] Jesenberger V, Jentsch S (2002). Deadly encounter: ubiquitin meets apoptosis. Nat Rev Mol Cell Biol.

[5] Kerscher O, Felberbaum R, Hochstrasser M (2006). Modification of proteins by ubiquitin and ubiquitin-like proteins. Annu Rev Cell Dev Biol.

[6] Liu YC (2004). Ubiquitin ligases and the immune response. Annu Rev Immunol.

[7] Liu J, Zhang S, Cao J (2014). Research progress of proteasome inhibition in targeted therapies for non-small cell lung cancer. Bull Med Postgrad.

[8] Chou CP, Huang NC, Jhuang SJ, Pan HB, Peng NJ, Cheng JT, Chen CF, Chen JJ, Chang TH (2014). Ubiquitin-conjugating enzyme UBE2C is highly expressed in breast microcalcification lesions. PLoS ONE.

[9] Palumbo A, Da Costa NM, De Martino M, Sepe R, Pellecchia S, de Sousa VP, Nicolau Neto P, Kruel CD, Bergman A, Nasciutti LE (2016). UBE2C is overexpressed in ESCC tissues and its abrogation attenuates the malignant phenotype of ESCC cell lines. Oncotarget..

[10] Chen S, Chen Y, Hu C, Jing H, Cao Y, Liu X (2010). Association of clinicopathological features with UbcH10 expression in colorectal cancer. J Cancer Res Clin Oncol.

[11] Zhao ZK, Wu WG, Chen L, Dong P, Gu J, Mu JS, Yang JH, Liu YB (2013). Expression of UbcH10 in pancreatic ductal adenocarcinoma and its correlation with prognosis. Tumour Biol J Int Soc Oncodev Biol Med.

[12] Rajkumar T, Sabitha K, Vijayalakshmi N, Shirley S, Bose MV, Gopal G, Selvaluxmy G (2011). Identification and validation of genes involved in cervical tumourigenesis. BMC Cancer.

[13] Kim WT, Jeong P, Yan C, Kim YH, Lee IS, Kang HW, Kim YJ, Lee SC, Kim SJ, Kim YT (2016). UBE2C cell-free RNA in urine can discriminate between bladder cancer and hematuria. Oncotarget.

[14] Okamoto Y, Ozaki T, Miyazaki K, Aoyama M, Miyazaki M, Nakagawara A (2003). UbcH10 is the cancer-related E2 ubiquitin-conjugating enzyme. Can Res.

[15] Parris TZ, Kovacs A, Aziz L, Hajizadeh S, Nemes S, Semaan M, Forssell-Aronsson E, Karlsson P, Helou K (2014). Additive effect of the AZGP1, PIP, S100A8 and UBE2C molecular biomarkers improves outcome prediction in breast carcinoma. Int J Cancer.

[16] Psyrri A, Kalogeras KT, Kronenwett R, Wirtz RM, Batistatou A, Bournakis E, Timotheadou E, Gogas H, Aravantinos G, Christodoulou C (2012). Prognostic significance of UBE2C mRNA expression in high-risk early breast cancer. A Hellenic Cooperative Oncology Group (HeCOG) Study. Ann Oncol Off J Eur Soc Med Oncol.

[17] Loussouarn D, Campion L, Leclair F, Campone M, Charbonnel C, Ricolleau G, Gouraud W, Bataille R, Jezequel P (2009). Validation of UBE2C protein as a prognostic marker in node-positive breast cancer. Br J Cancer.

[18] Shen Z, Jiang X, Zeng C, Zheng S, Luo B, Zeng Y, Ding R, Jiang H, He Q, Guo J (2013). High expression of ubiquitin-conjugating enzyme 2C (UBE2C) correlates with nasopharyngeal carcinoma progression. BMC Cancer.

[19] Pu X, Shi J, Li Z, Feng A, Ye Q (2015). Comparison of the 2007 and 2013 ASCO/CAP evaluation systems for HER2 amplification in breast cancer. Pathol Res Pract.

[20] Pastrez PRA, Mariano VS, da Costa AM, Silva EM, Scapulatempo-Neto C, Guimaraes DP, Fava G, Neto SAZ, Nunes EM, Sichero L (2017). The relation of HPV infection and expression of p53 and p16 proteins in esophageal squamous cells carcinoma. J Cancer.

[21] Iwamoto T, Katagiri T, Niikura N, Miyoshi Y, Kochi M, Nogami T, Shien T, Motoki T, Taira N, Omori M (2017). Immunohistochemical Ki67 after short-term hormone therapy identifies low-risk breast cancers as reliably as genomic markers. Oncotarget.

[22] Goldhirsch A, Wood WC, Coates AS, Gelber RD, Thurlimann B, Senn HJ (2011). Strategies for subtypes–dealing with the diversity of breast cancer: highlights of the St. Gallen International Expert Consensus on the Primary Therapy of Early Breast Cancer 2011. Ann Oncol.

[23] Uhlen M, Fagerberg L, Hallstrom BM, Lindskog C, Oksvold P, Mardinoglu A, Sivertsson A, Kampf C, Sjostedt E, Asplund A (2015). Proteomics. Tissue-based map of the human proteome. Science (New York, NY).

[24] Rhodes DR, Kalyana-Sundaram S, Mahavisno V, Varambally R, Yu J, Briggs BB, Barrette TR, Anstet MJ, Kincead-Beal C, Kulkarni P (2007). Oncomine 3.0: genes, pathways, and networks in a collection of 18,000 cancer gene expression profiles. Neoplasia (New York, NY).

[25] Rhodes DR, Yu J, Shanker K, Deshpande N, Varambally R, Ghosh D, Barrette T, Pandey A, Chinnaiyan AM (2004). ONCOMINE: a cancer microarray database and integrated data-mining platform. Neoplasia (New York, NY).

[26] Deng M, Bragelmann J, Kryukov I, Saraiva-Agostinho N, Perner S (2017). FirebrowseR: an R client to the Broad Institute’s Firehose Pipeline. Database.

[27] Gao J, Aksoy BA, Dogrusoz U, Dresdner G, Gross B, Sumer SO, Sun Y, Jacobsen A, Sinha R, Larsson E (2013). Integrative analysis of complex cancer genomics and clinical profiles using the cBioPortal. Sci Signal.

[28] Yu H, King RW, Peters JM, Kirschner MW (1996). Identification of a novel ubiquitin-conjugating enzyme involved in mitotic cyclin degradation. Curr Biol.

[29] Doherty FJ, Dawson S, Mayer RJ (2002). The ubiquitin-proteasome pathway of intracellular proteolysis. Essays Biochem.

[30] Rawat A, Gopal G, Selvaluxmy G, Rajkumar T (2013). Inhibition of ubiquitin conjugating enzyme UBE2C reduces proliferation and sensitizes breast cancer cells to radiation, doxorubicin, tamoxifen and letrozole. Cell Oncol (Dordrecht)..

[31] Shuliang S, Lei C, Guangwu J, Changjie L (2013). Involvement of ubiquitin-conjugating enzyme E2C in proliferation and invasion of prostate carcinoma cells. Oncol Res.

[32] Tang XK, Wang KJ, Tang YK, Chen L (2014). Effects of ubiquitin-conjugating enzyme 2C on invasion, proliferation and cell cycling of lung cancer cells. Asian Pac J Cancer Prev.

[33] Pan Z, Guo S, Wang C, Gu Y, Bao J (2014). Expression of UbcHlO correlates with prognosis of infiltrating ductal carcinoma of breast. J Surg Concepts Pract.

[34] Berlingieri MT, Pallante P, Sboner A, Barbareschi M, Bianco M, Ferraro A, Mansueto G, Borbone E, Guerriero E, Troncone G (2007). UbcH10 is overexpressed in malignant breast carcinomas. Eur J Cancer.

[35] Pan JY, Zhang F, Sun CC, Li SJ, Li G, Gong FY, Bo T, He J, Hua RX, Hu WD (2017). miR-134: a human cancer suppressor?. Mol Ther Nucleic Acids..

[36] Phuah NH, Azmi MN, Awang K, Nagoor NH (2017). Suppression of microRNA-629 enhances sensitivity of cervical cancer cells to 1′S-1′-acetoxychavicol acetate via regulating RSU1. Onco Targets Ther.

[37] Zhang Z, Song X, Tian H, Miao Y, Feng X, Li Y, Wang H (2017). MicroRNA-137 inhibits growth of glioblastoma through EGFR suppression. Am J Transl Res.

[38] Cervantes-Anaya N, Ponciano-Gomez A, Lopez-Alvarez GS, Gonzalez-Reyes C, Hernandez-Garcia S, Cabanas-Cortes MA, Garrido-Guerrero JE, Villa-Trevino S (2017). Downregulation of sorting nexin 10 is associated with overexpression of miR-30d during liver cancer progression in rats. Tumour Biol.

[39] Xiang T, Hu AX, Sun P, Liu G, Liu G, Xiao Y (2017). Identification of four potential predicting miRNA biomarkers for multiple myeloma from published datasets. Peer J..

[40] Chen WJ, Gan TQ, Qin H, Huang SN, Yang LH, Fang YY, Li ZY, Pan LJ, Chen G (2017). Implication of downregulation and prospective pathway signaling of microRNA-375 in lung squamous cell carcinoma. Pathol Res Pract.

[41] Yuan Y, Zhang H, Liu X, Lu Z, Li G, Lu M, Tao X (2017). MicroRNA signatures predict prognosis of patients with glioblastoma multiforme through the cancer genome atlas. Oncotarget..

[42] Zhang G, Zheng H, Zhang G, Cheng R, Lu C, Guo Y, Zhao G (2017). MicroRNA-338-3p suppresses cell proliferation and induces apoptosis of non-small-cell lung cancer by targeting sphingosine kinase 2. Cancer Cell Int.

[43] Huang S, Feng C, Zhai YZ, Zhou X, Li B, Wang LL, Chen W, Lv FQ, Li TS (2017). Identification of miRNA biomarkers of pneumonia using RNA-sequencing and bioinformatics analysis. Exp Ther Med.

[44] Song Y, Li J, Zhu Y, Dai Y, Zeng T, Liu L, Li J, Wang H, Qin Y, Zeng M (2014). MicroRNA-9 promotes tumor metastasis via repressing E-cadherin in esophageal squamous cell carcinoma. Oncotarget..

[45] Selcuklu SD, Donoghue MT, Rehmet K, de Souza Gomes M, Fort A, Kovvuru P, Muniyappa MK, Kerin MJ, Enright AJ, Spillane C (2012). MicroRNA-9 inhibition of cell proliferation and identification of novel miR-9 targets by transcriptome profiling in breast cancer cells. J Biol Chem.

[46] Gohring AR, Reuter S, Clement JH, Cheng X, Theobald J, Wolfl S, Mrowka R (2017). Human microRNA-299-3p decreases invasive behavior of cancer cells by downregulation of Oct4 expression and causes apoptosis. PLoS ONE.

[47] Huang YH, Liang KH, Chien RN, Hu TH, Lin KH, Hsu CW, Lin CL, Pan TL, Ke PY, Yeh CT (2017). A circulating MicroRNA signature capable of assessing the risk of hepatocellular carcinoma in cirrhotic patients. Sci Rep.

[48] Hu J, Xu Y, Cai S (2015). Specific microRNAs as novel biomarkers for combination chemotherapy resistance detection of colon adenocarcinoma. Eur J Med Res.

[49] Meng W, Tai Y, Zhao H, Fu B, Zhang T, Liu W, Li H, Yang Y, Zhang Q, Feng Y (2017). Downregulation of miR-33a-5p in hepatocellular carcinoma: a possible mechanism for chemotherapy resistance. Med Sci Monit Int Med J Exp Clin Res.

[50] Zhang Y, Li T, Qiu Y, Zhang T, Guo P, Ma X, Wei Q, Han L (2017). Serum microRNA panel for early diagnosis of the onset of hepatocellular carcinoma. Medicine..

[51] Zhang Y, Huang F, Wang J, Peng L, Luo H (2015). MiR-15b mediates liver cancer cells proliferation through targeting BCL-2. Int J Clin Exp Pathol.

[52] Dai L, Wang Y, Chen L, Zheng J, Li J, Wu X (2017). MiR-221, a potential prognostic biomarker for recurrence in papillary thyroid cancer. World J Surg Oncol.

[53] Zhang Y, Han T, Wei G, Wang Y (2015). Inhibition of microRNA-17/20a suppresses cell proliferation in gastric cancer by modulating UBE2C expression. Oncol Rep.

[54] Han Q, Zhou C, Liu F, Xu G, Zheng R, Zhang X (2015). MicroRNA-196a post-transcriptionally upregulates the UBE2C proto-oncogene and promotes cell proliferation in breast cancer. Oncol Rep.

